# Seeking gastroenterological services during a pandemic: lessons from a large, national, population-based survey during the COVID-19 pandemic

**DOI:** 10.1093/jcag/gwaf038

**Published:** 2026-01-13

**Authors:** Sachin Srinivasan, Sravanthi Parasa, Paul Sinclair, Kevin Kennedy, Gary Falk, David Armstrong, Prateek Sharma

**Affiliations:** University of Kansas School of Medicine, Kansas City, KS 66160, United States; Kansas City VA Medical Center, Kansas City, MO 64128, United States; Swedish Medical Center, Seattle, WA 98122, United States; INSINC Consulting Inc., Guelph, ON N1G 3G3, Canada; Kansas City VA Medical Center, Kansas City, MO 64128, United States; University of Pennsylvania, Philadelphia, PA 19104, United States; Division of Gastroenterology, McMaster University, Hamilton, ON, L8S 4L8, Canada; Farncombe Family Digestive Health Research Institute, McMaster University, Hamilton, ON, L8S 4K1, Canada; University of Kansas School of Medicine, Kansas City, KS 66160, United States; Kansas City VA Medical Center, Kansas City, MO 64128, United States

**Keywords:** COVID-19, SARS-CoV-2, gastrointestinal endoscopy, psychological barriers, patients, survey

## Abstract

**Background:**

The 2019 coronavirus pandemic (COVID-19) caused significant disruptions in people’s lives, healthcare-seeking behavior, and willingness to undergo endoscopic procedures.

**Methods:**

This large national survey of adults used an online platform to collect participants' de-identified demographics, attitudes, and opinions regarding healthcare-seeking behavior and endoscopy during the COVID-19 pandemic. Data were analyzed using descriptive statistics and multivariate logistic regression.

**Results:**

There were 29 449 respondents; mean age 43.3 ± 17.3 years, 72% female. Among 3928 respondents who visited their doctor virtually during the COVID-19 pandemic, most were satisfied (76%). In a multivariate analysis, respondents who were satisfied or neutral toward a virtual visit were more likely to be married, African American, and have some college education. In contrast, those who were dissatisfied were more likely to be older or female. Only 26.3% (*n* = 7746) reported concerns about undergoing endoscopy during the pandemic. Among those respondents, preferences were to reschedule 3-4 months later (38%) after having had the vaccine (19%) or to forgo the procedure entirely (28%). In a multivariate analysis, having had a prior endoscopy was most strongly associated with concern, followed by African American race, female sex, being married, and older age.

**Conclusion:**

This large national survey suggested high satisfaction with virtual visits and low concern about undergoing endoscopy during the pandemic. Concern around endoscopy was increased among those who had a prior endoscopy, African Americans, women, and older age groups.

## Introduction

COVID-19, the infectious disease caused by severe acute respiratory syndrome coronavirus-2 (SARS-CoV-2), was first identified in the United States in January 2020, after which the virus began to spread rapidly from person to person.^1^ By March 2020, the World Health Organization (WHO) declared COVID-19 a pandemic, statewide shutdowns began, and reimbursement for telehealth was expanded in the United States. By April 2020, there were more than 1 million cases worldwide, and by the end of May 2020, the death toll in the United States exceeded 100 000. States began to reopen in late April and May 2020. As of mid-2022, there were over 84 million cases and 1 million deaths due to COVID-19 in the United States. The first vaccine received emergency use authorization in the United States on December 11, 2020. As of June 2025, more than 777 million cases and 7 million deaths had been reported worldwide; 70% of the US population has now been fully vaccinated compared with about 55%-57% at the time of this survey, which was conducted between September 8 and 21, 2021.^2^^,^^3^

On September 11, 2020, data released by the Centers for Disease Control and Prevention showed that approximately 41% of adults in the United States delayed or avoided seeking medical care (urgent or nonurgent) due to COVID-19 concerns.^1^ The same report noted that individuals who were non-white Hispanic, Black unpaid caregivers for adults, suffering from an underlying medical condition or disability, or considered as young adults were among the most affected subgroups.^1^

A corresponding reduction in endoscopic procedures also occurred, with multiple international surveys of endoscopy units reporting cutbacks of more than 50% in procedure volume in the initial months of the pandemic.^4^^,^^5^ However, relatively little was known about patients' willingness to undergo endoscopy during the initial phases of the COVID-19 pandemic; as such, this survey was conducted to better elucidate patient perspectives relating to endoscopy at this time. In addition to reporting attitudes toward endoscopy at scale, this study uniquely couples those perceptions with real‑world timing preferences (deferral vs forgoing) and experiences with virtual care in a single, population‑based US sample.

## Methods

### Study design

This was a cross-sectional national survey of adults 18-85 years of age to assess knowledge, concerns, attitudes, and safety practices regarding endoscopic procedures, and virtual healthcare visits during the COVID-19 pandemic. Attitudes around vaccines were also collected but will be reported in a separate publication.

The survey comprised 50 questions, about sociodemographics and attitudes and opinions related to the COVID-19 pandemic, vaccine use, healthcare-seeking behavior, and endoscopic procedures. The survey was developed by a research team selected by the principal investigator (Supplement S1). No formal pretesting of the questionnaire was conducted. The instrument was designed to collect factual information rather than to generate composite scores and so pretesting was deemed not necessary. The results and discussion pertain to the survey’s healthcare-seeking behavior and endoscopy portion during the COVID-19 pandemic. The results from the study related to vaccine use and hesitancy are submitted as another publication by the same authors. The authors adhered to the Checklist for Reporting of Survey Studies guidelines as recommended by the EQUATOR Network. The completed checklist is available as Supplement S2.

### Study population and recruitment

After obtaining approval from the Swedish Medical Center Institutional Review Board, a representative national sample of the general population was invited via email. Potential participants were provided a link to the online survey and advised of an approximate completion time of 15 minutes. Utilizing Cint, a survey research company that employs a reward mechanism to motivate participants to engage in surveys, potential participants received an email inviting them to participate in an online survey. This email contained a standardized message that individual research panels could customize. It stated, “Based on the information in your [research panel] profile, we have a survey that matches your qualifications. The survey should take about 15 minutes, and upon completion, you will receive [incentive] credited to your account.” The survey’s length determines Cint’s reward system and requires criteria to be met before participants can redeem rewards. This setup fosters ongoing participation and discourages individuals who solely participate in surveys for financial gain. Cint maintains large, preexisting, opt‑in panels of adult respondents who have consented to be contacted for research; eligible panelists are invited via e‑mail based on profile attributes, and participation is voluntary with small incentives. Because the sampling frame is panel‑based, a traditional response rate could not be calculated.

The survey was conducted between September 8 and 21, 2021, using MyGIHealth, a mobile application utilizing the Automated Evaluation of GI Symptoms algorithm previously validated and used in other large nationwide population-based studies.^6^^,^^7^ The survey was circulated using the Cint (cint.com) survey platform. The sampling quotas were set to approximate the US adult population by age, sex, and region; however, representation of racial and ethnic minorities, lower socioeconomic status groups, and non‑English speakers was limited by use of English‑language, online‑only recruitment.

### Data collection and statistical analysis

All respondents were ≥18 years of age and assigned a unique identification number. No other patient identifiers were used or collected. Study data (including data queries) was managed using Microsoft Excel and PowerBI (Microsoft, Seattle, WA, United States). Descriptive statistics and multivariate logistic regression were used for data analysis. A 2-tailed *P*-value of less than .05 was considered statistically significant. We performed population-weighted multivariable regression models to adjust for potentially confounding factors and to calculate adjusted *P*-values, odds ratios (OR), and 95% confidence intervals (CI). We used logistic and linear multivariable regression models for binary and continuous outcomes.

## Results

A total of 29 449 surveys were included in the analysis. Surveys were excluded if the age entered was <18 or >85, incomplete forms, or had unanalyzable data (eg, unrelated text phrase response in place of numeric response). The mean age of respondents was 43.3 (±17.3) years. The majority were female (71.5%), Caucasian (74.5%), and had at least a high school education (98.9%). Additional demographic characteristics are shown in Table 1.

**Table 1:** Demographics of survey respondents (*N* = 29 449).

Characteristics	Respondents, *n* (%)
**Mean age, years [±SD]**	43.3 [17.3]
**Female sex**	21 064 (71.5)
**Marital status**	
** Married**	12 126 (41.2)
** Single**	11 667 (39.6)
** Divorced**	3405 (11.6)
** Widowed**	1470 (5.0)
** Separated**	781 (2.7)
**Race**	
** White**	21 944 (74.5)
** Black or African American**	4069 (13.8)
** Asian**	1358 (4.6)
** Hispanic or Latino**	817 (2.8)
** American Indian or Alaska Native**	557 (1.9)
** Bi/multiracial ethnicity**	345 (1.2)
** Native Hawaiian or other Pacific Islander**	260 (0.9)
** Prefer not to answer**	99 (0.3)
**Level of education**	
** Completed graduate school**	4108 (13.9)
** Graduated from college (undergraduate)**	7960 (27.0)
** 1-3 years of college**	8541 (29.0)
** High school**	8509 (28.9)
** Middle school**	331 (1.1)
**≥1 Child in household**	10 491 (35.6)
**≥1 Parent living with respondent^a^**	6805 (23.1)

a Do you have parents (either your or significant other’s) living with you at home?

### Healthcare-seeking behavior

**Figure 1:**
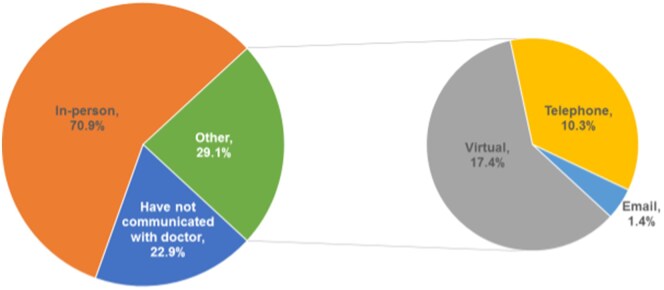
Type of visit among respondents who had seen or communicated with their doctor since the start of COVID-19 pandemic.

While almost two-thirds of respondents stated that contact with their doctor’s office was unchanged during the COVID-19 pandemic (62.2%; *n* = 18 310), 28.5% indicated a lower frequency of contact. Among those who had contact with their doctor since the onset of the COVID-19 pandemic, the majority (70.9%; *n* = 16 031) had an in-person office visit, while 27.7% had virtual or telephone contact (Figure 1).

Most of the 3928 respondents who had a virtual visit with their doctor since the COVID-19 pandemic began were satisfied (76%). Compared to an in-person visit, 39.7% rated the virtual visit better, 36.3% found no difference, and only 24.1% rated the virtual visit worse. In a multivariate analysis, respondents who were satisfied or neutral toward a virtual visit were more likely to be married, African American, or have some college education. In contrast, those dissatisfied with their virtual visit were more likely to be older or female (Table 2). Challenges with virtual visits were common (60% of respondents), with the most common being lack of in-person interaction (23.4%) and technical problems (18.4%). However, 74.2% of respondents would be willing to have a virtual visit again.

**Table 2:** Multivariate odds ratios for characteristics significantly associated with satisfaction^a^ or dissatisfaction with virtual visits during COVID-19 pandemic (*N* = 3928).

Characteristics	Odds ratio	95% CI
**Married versus any other marital status combined**	1.16	1.07-1.27
**African American versus any other race combined**	1.20	1.08-1.33
**Some college** ^b^ **versus no college**	1.31	1.21-1.42
**Older (per 10 years)**	0.84	0.82-0.86
**Female versus male**	0.92	0.85-1.00

a Satisfaction included respondents who chose “virtual visit was better” or “no difference” compared to an in-person visit.

b Includes: completed graduate school, graduated from college (undergraduate), and 1-3 years of college.

While 23.2% would be comfortable with an in-person visit in the future regardless of circumstances, 14.3% would remain uncomfortable regardless of COVID-19 protocols. Almost half (45.9%) of respondents would feel comfortable if appropriate COVID-19 protection protocols were in place.

### Attitudes around gastrointestinal endoscopy

In the overall group, almost 40% of respondents indicated that if they were due for an endoscopic procedure like screening, they were not worried about COVID-19 and would be willing to undergo it at any convenient time. In contrast, 26.3% (*n* = 7746) of respondents indicated that the COVID-19 pandemic had caused them concerns about undergoing a screening/nonemergent endoscopic procedure. Among this group of respondents, 38% preferred to reschedule the procedure in 3-4 months, 28% would forgo the procedure entirely, and 19% would reschedule the procedure after receiving a COVID-19 vaccine.

In this group, 34% of respondents would be more comfortable undergoing endoscopy if staff were masked and donned other Personal Protective Equipment (PPE), and 17% would be more comfortable if they and the endoscopy staff were tested for COVID-19, but 36% stated that nothing would make them comfortable enough to do so.

In a multivariate analysis, having had a prior endoscopy was most strongly associated with concern regarding undergoing endoscopy during the COVID-19 pandemic (Table 3). Female sex, older age, being married, and African American race were also associated with concerns.

**Table 3:** Multivariate odds ratios for characteristics significantly associated with “concern” for undergoing endoscopy during COVID-19 pandemic (*N* = 7746/29 449).

Characteristics	Odds ratio	95% CI
**Prior endoscopy**	1.88	1.78-2.00
**African American versus any other race combined**	1.45	1.34-1.57
**Female versus male**	1.16	1.09-1.23
**Married versus any other marital status combined**	1.07	1.00-1.14
**Older (per 10 years)**	1.07	1.05-1.09

## Discussion

In this large national US survey-based study conducted during the COVID-19 pandemic, among the 27.7% of respondents who had a virtual visit, approximately 3 in 4 reported being comfortable with ongoing virtual care; the majority of respondents continued in-person care during the same period. Only 1 in 4 of the individuals had some concern about undergoing any endoscopy during a pandemic; of those, only a quarter of the concerned individuals would forgo endoscopy altogether (instead of deferring to later or after vaccination). These results suggest that individuals are more receptive to virtual care when necessary and are willing to undergo endoscopic procedures even during a pandemic if recommended by their providers. Our findings are consistent with gastrointestinal (GI)-specific surveys^8–11^ reporting high satisfaction with telehealth among patients and providers and variable access during the pandemic; they align with patterns showing younger and female patients more frequent users in some cohorts and highlight equity considerations for older adults and those with limited digital resources.

A study of 318 consecutive patients undergoing endoscopy-related telehealth appointments compared no-show rates to a historical sample of 373 in-person clinic visits during the same period in 2019.^12^ Telehealth consults reduced no-shows by approximately 50% (6.4% vs 12.6%; *P* < .01).

Among endoscopic procedures, a retrospective survey^13^ (*n* = 847; 13 GI units) in Italy of fast-tracked (due within 10 working days) endoscopic procedures (urgent endoscopy) during 3 weeks in March 2020 (initial period of COVID-19) showed that the no-show rate was 29.4% (*n* = 249) overall and increased progressively over the 3 weeks (week 1 = 15.1%; week 2 = 29.6%; week 3 = 48.2%), whereas no-shows had never exceeded 2% during the same time in the prior year. Another prospective, observational study^14^ found significantly lower rates of colonoscopy and Fecal immuochemical Test (FIT; a non-invasive stool based screening for colon cancer) screening and higher rates of cancellation/rescheduling for December 2019-April 2020 (vs same period for prior 3 years). Among patients canceling or postponing their appointment, 7 of 14 patients (50%) did so due to COVID-19 infection fears, whereas no patients in the preceding 3 years rescheduled/canceled because of infection fears.

Another prospective, single-center study to assess COVID-19 exposure concern, perception of strategies taken by the center to mitigate COVID-19 risk during endoscopy, and patient satisfaction in 2 cohorts showed older age (OR 1.02; 95% CI, 1.01-1.04), non-white ethnicity (OR 2.5; 95% CI, 1.5-4.3), and screening-based indication for GI endoscopy (OR 1.7; 95% CI, 1.1-2.9) were all associated with a “high” level of concern of COVID-19 exposure.^15^

The study has its strengths and limitations. This study is the largest national survey on attitudes to clinic visits and endoscopy during COVID-19, with over 29 000 respondents. The study was performed to seek the perceptions of people undergoing GI endoscopy and virtual care during the COVID-19 pandemic. While some of the comparative outcomes are subtle, the large sample size allowed us to have the power to detect and report any statistically significant difference. The timing of the survey is key to note, as this was when vaccinations became available and were adopted across the country. Earlier studies showed some hesitancy with doctor visits and endoscopy, but with better hygiene practices, use of PPE, and vaccination, this survey showed an increased willingness to undergo endoscopic procedures and possibly improve day-to-day healthcare visits overall. The study has its limitations. This was an online survey study, and online data collection does raise the concern of generalizability, especially to elderly individuals and those without access to computers, smartphones, or internet access. While our study certainly had older individuals, it could have been selected for those more independent and functional. First, because recruitment used an online, opt‑in panel, a conventional response rate could not be computed, and nonresponse bias cannot be excluded; respondents may differ systematically from nonrespondents in access to technology, health status, or health‑seeking behavior.

Additionally, the study evaluated participants' perceptions. Although respondents' opinions may be amenable to change, this study cannot determine the effect of an individual’s opinions on their behavior or whether actions to change their opinions would produce a measurable effect on participants' willingness to adopt virtual healthcare visits or attend endoscopic procedures. However, this information still provides insight into individuals' overall thinking and behavior, which is key to preparing for any future eventualities.

In conclusion, this large national survey suggested satisfaction with virtual visits and low concern about undergoing endoscopy during the pandemic. Certain gender, race, and education differences impact an individual’s level of concern or comfort in undergoing endoscopy. An awareness campaign addressing these groups with targeted follow-up surveys could shed light on changes in perceptions and potential interventions to reduce concern and improve participation in gastrointestinal care not only during adversities but in general as well.

## Supplementary Material

gwaf038_Supplementary_Data

## Data Availability

Data are available on request to the corresponding author.
