# Supplementary material for: Seeking gastroenterological services during a pandemic: lessons from a large, national, population-based survey during the COVID-19 pandemic
Source: J Can Assoc Gastroenterol. 2026 Jan 13;9(2):129–33. doi: 10.1093/jcag/gwaf038 (PMC13123699; doi:10.1093/jcag/gwaf038)
Supplement: gwaf038_Supplementary_Data [file gwaf038_supplementary_data.zip › Supplement 1.docx]

# Supplement 1 – Survey Questionnaire

**Basic Demographics**

1. Age ___ *(in years)*
2. Gender: (*male/female*)
3. City (*free text response/zip code if only US based survey*)
4. Marital status (*married/widowed/separated/divorced/single*)
5. Do you have children at home? (*Yes/No*)
6. Do you have parents (either your or significant other’s) living with you at home? (*Yes/No*)
7. Are you of Hispanic, Latino, or of Spanish origin? (*Yes/No*)
8. How would you describe yourself? (*American Indian or Alaska Native/Asian/Black or African American/Native Hawaiian or Other Pacific Islander/White*)
9. What is the highest level of education? (*Middle school/High school/1-3 years of college/Graduated from College (undergraduate)/Completed Graduate school*)
10. Co-morbidities and current medication:
    1. Co-morbidities (*Choose all that apply – Gastro-esophageal reflux disease, Inflammatory bowel disease , Eosinophilic Esophagitis, Irritable bowel syndrome , Celiac disease, h/o colon polyps, h/o GI cancer, chronic liver disease, chronic heart disease, chronic kidney disease. chronic lung disease, diabetes, hypertension, high cholesterol*)
    2. Choose all that apply for the groups of medications you are on:

- *acid suppressive medications like pantoprazole/omeprazole/rabeprazole/lansoprazole;*
- *antacids;*
- *steroids;*
- *biologic agents (infliximab, adalimumab, vedolizumab);*
- *disease modifying agents like mesalamine, azathioprine;*
- *BP lowering medications;*
- *oral diabetic medications, injectable diabetes medications other than insulin, insulin;*
- *nebulized/inhaled medications – albuterol/budesonide-formoterol/tiotropium*
- *cholesterol lowering medications – atorvastatin/rosuvastatin/lovastatin/pravastatin, fish oil, fenofibrate*

1. Have you had any exposure to a COVID-19 infected person? (*Yes/No/Don’t know*)
2. Have you had any symptoms suggestive of COVID-19 infection since the beginning of this pandemic in 2020 (Yes/No)? **If No, skip to question 15.**
3. If yes, were you tested for COVID-19 infection (*Yes/No*)
4. If yes, did you test positive for COVID-19 infection (*Yes/No*)

**Knowledge and source of information regarding the pandemic**

1. Where do you get guidance for your behavior concerning the COVID-19 infection from (Please rank these in order of their importance (*most important to least important*) to you):

- *Television/News Sites/Newspaper*
- *Blogs and forums / Social Media*
- *Friends/Family*
- *Physician.*
- *Other, please specify____*

1. In your opinion, what percentage of people who get the COVID-19 infection, end up dying from it

(*0-5%, 10-20%, 50-60%, 90-100%)*

1. What percentage of people who get the influenza (Flu) do you think die from it? *(<1%, 5-10%, 50-60%, 80-90%*)
2. How would you rank the modalities (from most effective to least effective) in their ability to prevent transmission of COVID-19 virus?

- *Hand Washing*
- *Facial Masks*
- *Disinfection of Surfaces*
- *Avoid close contacts*

1. Do you think a vaccine will be able to prevent COVID-19 infection? (*Yes/No/I don’t know*)
2. How would you rank the age group in order of susceptibility (more susceptible to least susceptible) to COVID-19 infection?

- *<18*
- *18-35*
- *35-50*
- *50-75*
- *Over the age of 75*

**Attitudes and concern related to COVID-19 in general**

1. Are you concerned about getting infected with COVID-19? (*slider from 0-100; 0-not concerned at all to 100-I am so concerned that it affects everything I do*)
2. Are you concerned that you would die if you get the COVID-19 infection (*slider from 0-100; 0-not concerned at all to 100-I am so concerned that it affects everything I do*)?
3. Are you concerned that you would get COVID-19 infection in the supermarket or other public places (*slider from 0-100; 0-not concerned at all to 100-I am so concerned that it affects everything I do*)?
4. If recommended by a healthcare provider/entity, are you willing to have a COVID-19 test ? (*Yes/No/Don’t know*)
5. If no, why? (*Don’t believe the test is accurate/I don’t want to know the result/I have heard that it hurts sometimes/I am concerned about getting infected from the test itself*)

**Attitudes and concern related to COVID-19 and healthcare**

1. Are you concerned that the COVID-19 pandemic will have an impact on the treatment or follow-up of your medical condition? (*Yes/No/Don’t know*)
2. If yes to above, please use the slider to let us know the extent to which the following reasons might be making you concerned (*0-not concerned about this at all to 100-I am so concerned that it affects everything I do*)
   1. Your appointments/investigations in the hospital might be cancelled
   2. Your appointments/ investigations in the hospital are changed to a telephone or digital appointment
   3. Your treatment is delayed
   4. Your treatment is cancelled
3. In the last 3 YEARS, have you seen a doctor? (*Yes/No*)
4. **If Yes**, what was the reason? – (*routine physical/I have chronic problems I need to see my doctor regularly/I was sick*)
5. How concerned are you regarding getting infected with COVID-19 if you were to visit the hospital? (*slider from 0-100; 0-not concerned at all to 100-I am so concerned that it affects everything I do*)
6. How concerned are you regarding getting infected with COVID-19 if you were to visit the doctor’s office? (*slider from 0-100; 0-not concerned at all to 100-I am so concerned that it affects everything I do*)
7. How has the COVID-19 pandemic influenced the frequency of your contact with your doctor’s office?

*a. You contact them less frequently than usual*

*b. You contact them more frequently than usual*

*c. nothing has changed*

*d. You have stopped contacting the doctor’s office*

1. Have you seen or communicated with your doctor since the start of COVID-19 pandemic? (*Yes/No*) If No, skip to 37?
2. If yes, how? (*Telephone/Virtual visit/In-person/Discussed over email*)
3. Compared to the in-person visit in the doctor’s office, how would you rate your virtual (telephone/video call) visit with your doctor?
   1. *Virtual visit was better*
   2. *Virtual visit was worse*
   3. *No difference*
4. What are the challenges you have faced during a virtual visit?
   1. *Technical problems like connectivity, internet*
   2. *You feel the doctor/provider spent less time*
   3. *You feel that the In-person interaction was missing*
   4. *You feel it’s difficult to get prescriptions and refills in time*
   5. *You did not have any challenges*
5. Would you prefer a virtual visit to an in-person visit in the future?
   1. *Yes*
   2. *No*
   3. *Either is fine*
6. For a future in-person visit, which of the following would make you most comfortable to go into your doctor’s office?
   1. *Nothing can make you comfortable in this situation*
   2. *If you got tested for COVID-19*
   3. *If you and the doctor’s office staff were tested for COVID-19*
   4. *Everyone in the doctor’s office was wearing masks and appropriate protection*
   5. *You would go regardless of anything*
   6. *Would you be willing to undergo screening procedures (such as colonoscopy and breast mammograms (for females))*

**Attitudes and concern related to COVID-19 and GI endoscopy procedures**

1. Have you EVER had a Gastroenterology endoscopy procedure like upper endoscopy (EGD) or a colonoscopy? (*Yes/No*)
2. Due to the COVID-19 pandemic, are you concerned about undergoing a screening/non-emergent endoscopic procedure? (*Yes/No*)
3. Assuming you are due for an endoscopic procedure like screening colonoscopy, what would your preference of the timing be?
   1. *Anytime convenient, not worried about COVID-19*
   2. *Reschedule to 3-4 months from now*
   3. *Reschedule after I have got the vaccine*
   4. *You don’t want the procedure at all*
4. How important is it to you to get your COVID-19 testing done before your endoscopic procedure? (*slider – 0-not important at all to 100 – the most important thing*)
5. Which of the following would make you more comfortable to get an endoscopic procedure?
   1. *Nothing can make you comfortable in this situation*
   2. *If you got tested for COVID-19*
   3. *If you and the doctor’s office staff were tested for COVID-19*
   4. *Everyone in the doctor’s office was wearing masks and appropriate protection*
   5. *You would go regardless of anything*
6. What are your concerns regarding undergoing an endoscopic procedure?
   1. *You have NO concerns- you will get it done if you have to*
   2. *Getting the COVID-19 infection*
   3. *Spreading the COVID-19 infection (in case you had it)*
   4. *Both getting it and spreading it)*
7. Could you please rank the following based on its importance to you (*most important to least important*) for an endoscopy procedure.
   - Your driver is asked to drop you off and not enter the building
   - Your driver is allowed to enter the building and is asked to stay in a single person cubicle in the waiting room that is protected and cleaned with disinfectant after each use
   - You are provided with a surgical mask as soon as you reach registration and wear it for your entire stay
   - All staff wear surgical masks at all times during your stay
   - All staff while wearing gloves and masks stay at least 6 feet from you except when required
   - You have received a COVID-19 test prior to the procedure
   - All staff are tested for COVID-19 infection
8. If your top 3 measures above were ensured, would you feel comfortable to undergo the endoscopy procedure? (*Yes/No/Don’t know*)

**Attitudes and concern related to COVID-19 vaccine**

1. Would you take the COVID-19 vaccine when it becomes available?
   1. *Yes*
   2. *Maybe I don’t know*
   3. *No*
2. If No to 47, why?
   1. *It doesn’t work*
   2. *I could get COVID-19 from it*
   3. *I don’t believe in vaccines*
   4. *I want more data before I commit to it*
   5. *I am worried the vaccine might not cover all the strains of the virus*
3. If Yes to 47, rate the following based on why you want to take the vaccine (*most important to least important)*
   - *My doctor told me to take it*
   - *I can get the infection and die if I don’t take it*
   - *I can travel again*
   - *I can see my family again*
   - *I can go back to my favorite restaurant*
   - *I can get back to my normal life*
4. If you have children (<18 years of age), would you get them vaccinated when vaccine is available?
   1. *Yes*
   2. *No*
   3. *Not sure*
   4. *I do not have children (<18 years of age)*
